# Ultrasound Assessment of Autonomous Thyroid Nodules before and after Radioiodine Therapy Using Thyroid Imaging Reporting and Data System (TIRADS)

**DOI:** 10.3390/diagnostics10121038

**Published:** 2020-12-02

**Authors:** Simone Agnes Schenke, Jan Wuestemann, Michael Zimny, Michael Christoph Kreissl

**Affiliations:** 1 Division of Nuclear Medicine, Department of Radiology and Nuclear Medicine, University of Magdeburg, Leipziger Strasse 44, 39120 Magdeburg, Germany; jan.wuestemann@med.ovgu.de (J.W.); michael.kreissl@med.ovgu.de (M.C.K.); 2 Institute for Nuclear Medicine Hanau/Giessen/Frankfurt/Offenbach, Nussallee 7, 63450 Hanau, Germany; zimny@nuk-hu.de

**Keywords:** autonomously functioning thyroid nodule (AFTN), scintigraphy, TIRADS, radioiodine treatment, thyroid ultrasound

## Abstract

The Thyroid Imaging and Reporting System (TIRADS) allows a sonographic assessment of the malignancy risk of thyroid nodules (TNs). To date, there is a lack of systematic data about the change in ultrasound (US) features after therapeutic interventions. The aim of this study was to characterize the changes in autonomously functioning thyroid nodules (AFTNs) after radioiodine therapy (RIT) by using TIRADS. We retrospectively assessed data from 68 patients with AFTNs treated with RIT between 2016 and 2018 who had available first and second follow-up US imaging. Before RIT, 69.1% of the AFTNs were classified as low-risk TNs when applying Kwak TIRADS (EU-TIRADS 52.9%), 22.1% were intermediate-risk TNs (EU-TIRADS 19.1%), and 8.8% were high-risk TNs (EU-TIRADS 27.9%). Twelve months after RIT, 22.1% of the AFTNs showed features of high-risk TNs according to Kwak TIRADS (EU-TIRADS 45.6%). The proportion of intermediate TNs also increased to 36.8% (EU-TIRADS 29.4%), and 41.2% were low-risk TNs (EU-TIRADS 25%). A significant percentage of AFTNs presented with features suspicious for malignancy according to TIRADS before RIT, and this number increased significantly after therapy. Therefore, before thyroid US, thorough anamnesis regarding prior radioiodine treatment is necessary to prevent unneeded diagnostic procedures.

## 1. Introduction

Autonomously functioning thyroid nodules (AFTNs) appear as hyperfunctioning on ^99m^TcO_4_^−^ (^99m^Tc-pertechnetate) scintigraphy with increased uptake of the tracer. AFTNs present either as toxic or nontoxic depending on their effect on peripheral thyroid hormone levels. Although AFTNs are benign in approximately 96–99% [1,2] of cases, they may have a variable appearance in ultrasound (US) imaging. Of note, AFTNs are known to show suspicious US features in a substantial number of cases mimicking thyroid cancer [3,4].

Radioiodine therapy (RIT) represents a well-established and effective functional treatment for AFTNs. In Germany, RIT is performed after standardized radioiodine uptake measurements (“radioiodine testing”) to calculate the individual radioactivity dose for treatment. Beta-emitting iodine I-131 strongly accumulates in the thyroid after oral or intravenous administration. Intracellular uptake is facilitated by the sodium–iodide symporter [1,5,6]. Post-treatment control via thyroid scan, analysis of peripheral thyroid hormone levels, and US imaging are applied three to six months after RIT to assess treatment success [7,8]. Typical observations in US follow-up (FU) are the shrinkage of the AFTNs’ total volume [9,10,11], and changes in morphological features, such as echogenicity, have also been reported [12]. To date, one study has addressed US findings after RIT in Graves’ disease [13]. However, detailed data are sparse about changes in US features of AFTNs after RIT.

The Thyroid Imaging Reporting and Data System (TIRADS) can be used to perform standardized risk stratification of thyroid nodules (TNs) with US. TIRADS was initially introduced in congruence with an established system for breast imaging assessment (BIRADS) [14]. The fixed number of easily accessible features on imaging allow the likelihood of malignancy of TNs to be categorized. Additionally, TIRADS provides a standardized description for the clinical communication between different specialist groups. During the last decade, multiple reporting systems for US imaging of TNs have been introduced and have gradually gained acceptance in the clinical setting. All of these systems are based on the number and combination of certain US characteristics, such as echogenicity, composition, calcifications, margins, and shape. A recommendation for or against fine-needle aspiration cytology (FNAC) can then be made depending on the risk class and size of the TNs. The most common systems used to date are Kwak-TIRADS, EU-TIRADS, and ACR TI-RADS [15,16,17].

In this study, we applied Kwak- and EU-TIRADS to assess changes in US features of AFTNs after RIT, both at an earlier time point after 6 months and later, after 12 months.

## 2. Materials and Methods

This study was approved by the local ethics committee of the Magdeburg University Hospital (No. 36/19 RAD 365, approval date 05 March 2019) and conducted in concordance with the Helsinki Declaration. Due to the retrospective study design, the obligation to obtain informed consent was waived. For this study, an initial number of 222 consecutive patients were assessed at two study sites in Germany (Practice of Nuclear Medicine Hanau and Magdeburg University Hospital) between January 2016 and February 2018 (the date of radioiodine testing). A radioiodine uptake test was performed according to the German guideline for dosimetric purposes before RIT [7] and in order to exclude “trapping-only nodules”. The inclusion criteria were RIT of a solitary AFTN identified by ^99m^TcO_4_^−^ scintigraphy, pretherapeutic US not longer than two weeks before RIT, and dual time-point follow-up data (FU1 and FU2) including B-mode US imaging. The exclusion criteria were insufficient follow-up data and ambiguous correlation of US and scintigraphy. Finally, we included 68 patients with toxic and nontoxic AFTNs.

Thyroid US was performed by different investigators with at least three years of experience in thyroid US. Each device was equipped with a linear probe with a frequency of 6–15 MHz (Hanau: GE Logiq S7 Expert; Magdeburg GE: Logiq S9; both from GE Medical Systems Information Technologies GmbH, Freiburg, Germany). For our study, we decided to use two different kinds of US risk stratification systems. The Kwak-TIRADS is a pattern-based system whereas the EU-TIRADS is a weighted pattern-based method. TIRADS classification of the AFTNs with EU-TIRADS and Kwak-TIRADS was retrospectively performed by one experienced investigator (S.S.), blinded to the timepoints of the US images. Additionally, the size and volume of each TN and the location within the lobe were documented and correlated with the result of the thyroid scan performed before RIT.

The EU-TIRADS high-risk category of TNs is based on four US features indicative of possible malignancy (non-oval/round shape, irregular margins, microcalcifications, and marked hypoechogenicity). Provided that a TN shows only one of these specific features, it is immediately classified as EU-TIRADS 5 with a risk of malignancy of 26–87% (high-risk TNs). Anechoic or entirely spongiform TNs and entirely isoechoic or hyperechoic TNs are classified as EU-TIRADS 2 and 3, respectively, indicating a low risk of malignancy (0% and 2–4%, respectively, low-risk TNs). A mildly hypoechoic TN without suspicious features is categorized as EU-TIRADS 4 (6–17%, intermediate-risk TNs) [16].

The Kwak-TIRADS score is calculated by summing the number of suspicious US characteristics present in the TN of interest (solid or mostly solid nodule, hypoechogenicity, irregular margins, presence of microcalcifications, and a taller-than-wide shape/configuration). The risk class increases gradually with the number of applicable suspicious features. In detail, the following scores are assigned: TIRADS 3, no detected suspicious features (malignancy risk 1.7%); TIRADS 4A, one detected suspicious feature (malignancy risk 3.3%); TIRADS 4B, two suspicious features detected on US (malignancy risk 9.2%); TIRADS 4C, three or four suspicious features (malignancy risk 44.4–72.4%); and TIRADS 5, five detected suspicious features (malignancy risk 87.5%) [15]. In accordance with previously published data from our group, we defined the following risk classes: TIRADS 3 and 4A: low-risk TNs; TIRADS 4B: intermediate-risk TNs; and TIRADS 4C and 5: high-risk TNs [18,19].

Descriptive statistical parameters are expressed as the mean ± standard deviation (SD) or median and interquartile range (25th to 75th percentile) depending on whether the parameter was normally distributed. Normal distribution was confirmed or disconfirmed using visual assessment of the distribution and the Shapiro–Wilk test. The Mann–Whitney U-test, Wilcoxon’s rank sum test, and *t*-test were used for statistical analyses as indicated (Statistic Language R, version 3.4.4, Vienna, Austria and commercial Winstat^®^, version 2012.1.0.96, 2017, R. Fitch Software, Bad Krozingen, Germany). To compare TIRADS scores before RIT and during follow-up, we applied the paired Wilcoxon’s rank sum test. Changes in the categorical attribution of a particular US feature were assessed using the McNemar’s chi-square test. In all cases, statistical significance was assumed at *p*-values < 0.05.

## 3. Results

We included 68 patients (39 females, 63.0 ± 10.3 years old and 29 males, age 58.9 ± 14.5 years; *p* = 0.141) with 68 AFTNs. The clinical characteristics of both study sites are summarized in Table 1. Fifty-two percent of the AFTNs were located in the right lobe, 41% in the left lobe, and 7% in the thyroid isthmus (*p* = 0.167). The median activity administered for RIT was 539 Megabecquerel (MBq) (25th/75th-P: 347/788 MBq), and the achieved dose in the AFTNs was 362 Gy (25th/75th-P: 202/422 Gy). Before RIT, 70.6% of all patients had received no thyroid-specific medications (i.e., levothyroxine or antithyroid drugs), and of those, 60.4% showed suppressed TSH values below 0.2 mU/L. The median time for FU1 was 5 months (25th/75th-P: 3.6/6.1 months) and was 16 months (25th/75th-P: 12.4/19.8 months) for FU2. The maximum size of the AFTNs decreased significantly after RIT from a median of 33 mm (25th/75th-P: 27/36 mm, pretherapeutic) to 24 mm (25th/75th-P: 18.3/27.0 mm, FU1, *p* < 0.0001) and 20 mm (25th/75th-P: 14.0/26.5 mm, FU2, *p* < 0.0001).

Before RIT, AFTNs were predominately solid (47%) or partially cystic (47%), isoechoic (69%), wider than tall (84%), well circumscribed (90%), and showed no calcifications (78%, Table 2). After RIT, AFTNs appeared more often solid (FU1 56%, *p* = 0.09; FU2 60%, *p* = 0.28), less isoechoic (FU1 52%, *p* = 0.05; FU2 44%, *p* = 0.003), less well circumscribed (FU1 79%; FU2 66%), and more often blurred (FU1 10%, *p* = 0.08; FU 2 27%, 0.002). Calcifications were more common after RIT (FU1 28%, *p* = 0.23; FU2 35%, *p* = 0.37), as shown in Table 2. No change in shape was observed during FU.

**Figure 1:**
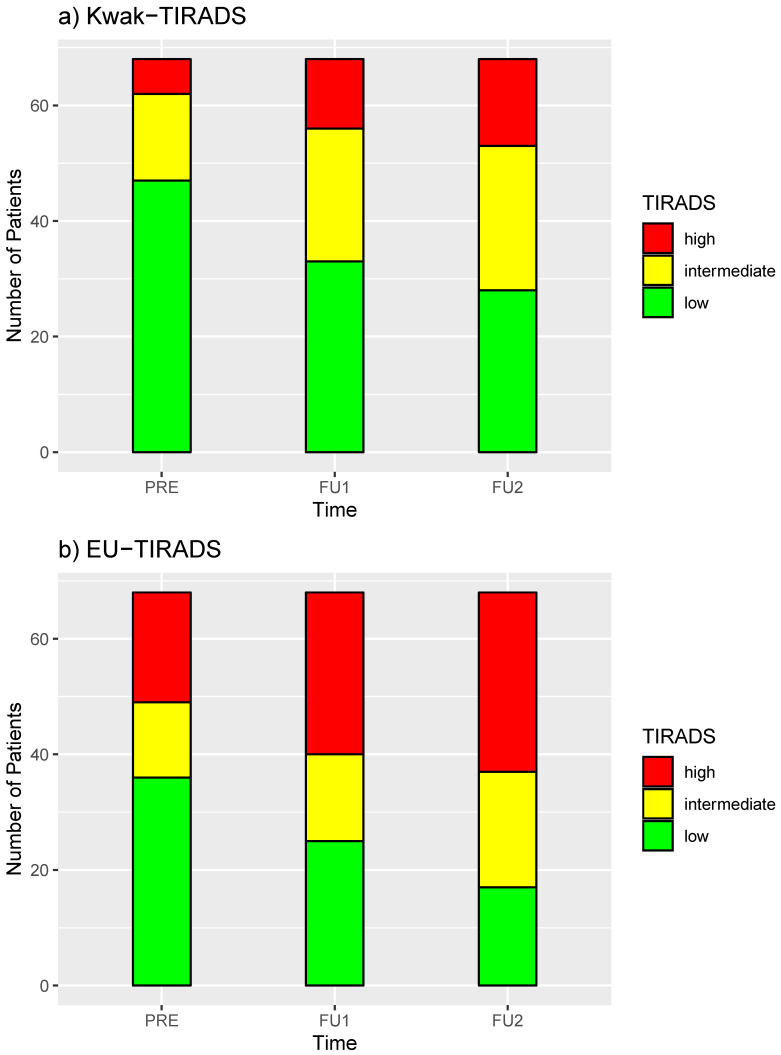
Relative frequency distribution (%) of the TIRADS categories before and after radioiodine therapy (Follow-up 1: FU1 and follow-up 2: FU2), (**a**) Kwak-TIRADS; (**b**) EU-TIRADS.

**Figure 2:**
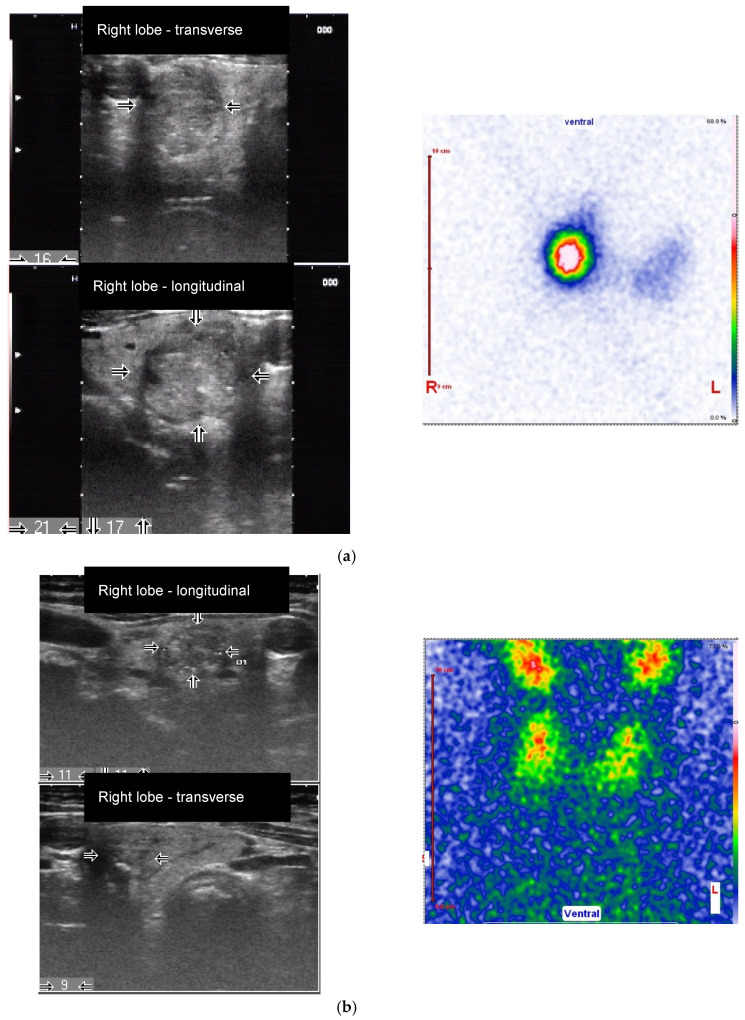

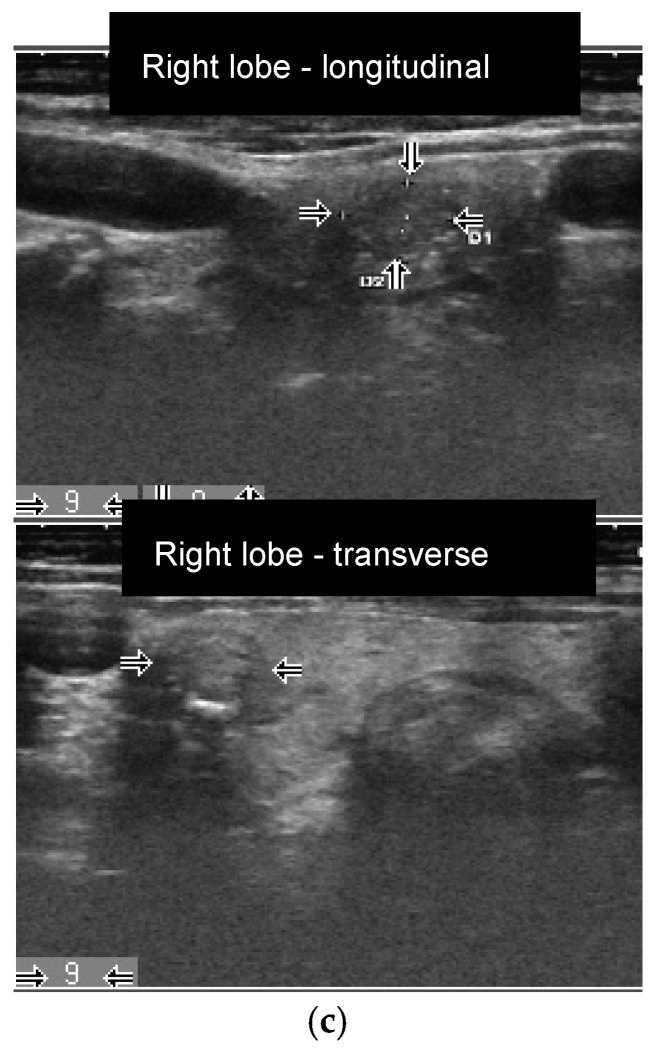
Female patient (77 years old) with an autonomously functioning thyroid nodule (AFTN) (^99m^Tc-pertechnetate image) in the right lower lobe (longitudinal and transverse images, nodule size: 16 × 17 × 21 mm, volume: 2.9 mL, arrows). (**a**) Before radioiodine therapy (RIT) (2016, TSH value < 0.1 mU/L, suppression with Levothyroxine) with 498 MBq (510 Gy). TIRADS classification: Kwak-TIRADS 4C (solid composition, microcalcifications, taller-than-wide shape, irregular margins); EU-TIRADS 5 (microcalcifications, taller-than-wide shape, irregular margins); (**b**) The same patient 7 months (follow-up 1, FU1) after RIT. The thyroid nodule size decreased to 9 × 11 × 11 mm (volume: 0.5 mL, arrows). Scintigraphy with ^99m^Tc-pertechnetate shows a hypofunctioning area in the lower right lobe, which reflects a therapeutic response (TSH value 1.2 mU/L, no thyroid medications). TIRADS classification: Kwak-TIRADS 4C (solid composition, isoechoic/hypoechoic parts, microcalcifications and macrocalcification, taller-than-wide shape); EU-TIRADS 5 (microcalcifications, taller-than-wide shape); (**c**) The same patient 12 months after RIT (TSH value 1.2 mU/L, no thyroid medications). The thyroid nodule size at FU2 was 9 × 8 × 9 mm (volume: 0.3 mL, −90% reduction, arrows). TIRADS classification: Kwak-TIRADS 4C (solid composition, mildly hypoechoic, microcalcifications and macrocalcification); EU-TIRADS 5 (microcalcifications).

**Figure 3:**
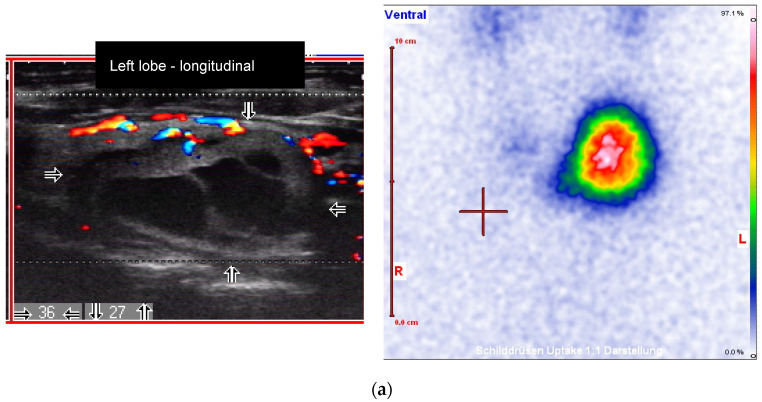

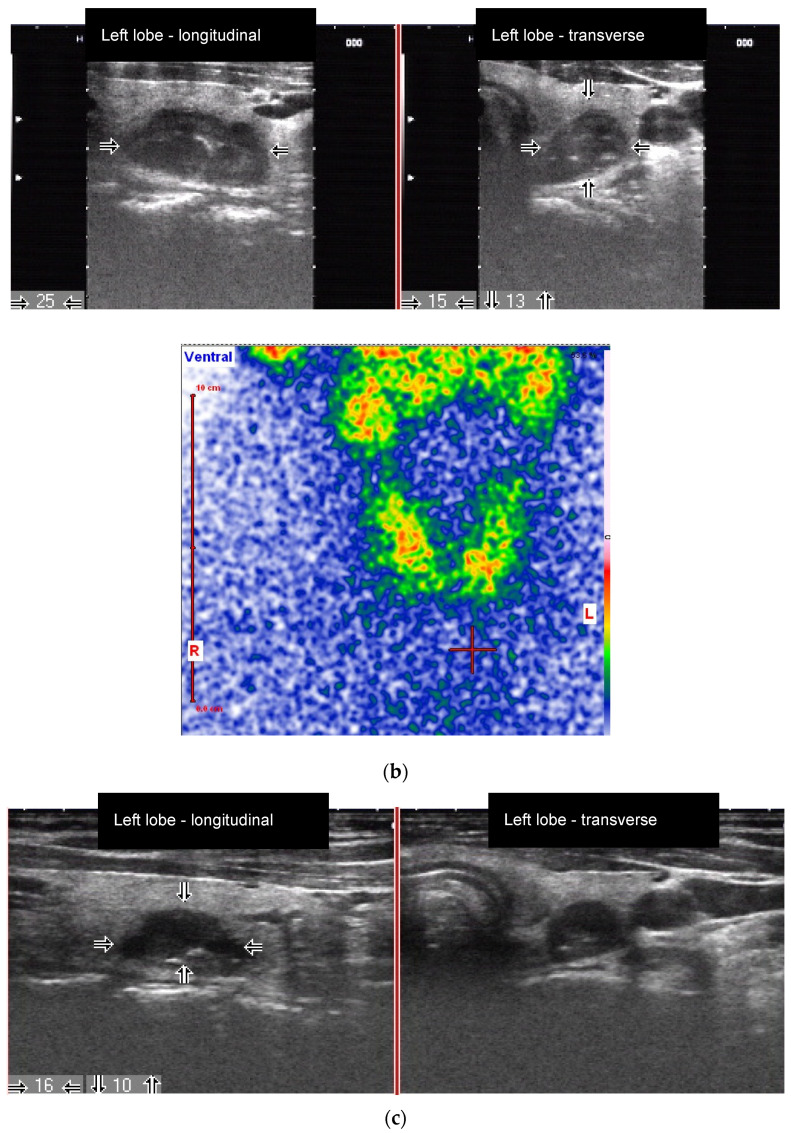
Male patient with an autonomously functioning thyroid nodule (AFTN) (scintigraphy with ^99m^Tc-pertechnetate) in the left thyroid lobe (25 × 27 × 36 mm; nodule volume 12.2 mL, arrows). (**a**) Before radioiodine therapy (RIT) (2016, TSH value 0.06 mU/L, no thyroid medications) with 1033 MBq (206 Gy due to reduced half-time at therapy). TIRADS classification: Kwak-TIRADS 4A (taller-than-wide shape); EU-TIRADS 5 (taller-than-wide shape); (**b**) The same patient 6 months after RIT (follow-up 1, FU1). The thyroid nodule size decreased to 15 × 13 × 25 mm (volume: 2.4 mL, arrows). Scintigraphy with ^99m^Tc-pertechnetate shows a non-hyperfunctioning area in the left lobe. TIRADS classification: Kwak-TIRADS 4C (predominantly solid composition, hypoechoic, microcalcifications); EU-TIRADS 5 (microcalcifications); (**c**) The same patient 12 months after RIT (TSH value 1.4 mU/L, Levothyroxine 50 micrograms/day). The thyroid nodule size at FU2 was 10 × 16 mm (arrows). TIRADS classification: Kwak-TIRADS 4C (solid composition, markedly hypoechoic, microcalcifications and macrocalcification); EU-TIRADS 5 (marked hypoechogenicity, microcalcifications).

Before RIT, 47% of all the AFTNs were categorized in the intermediate-risk (4: 19.1%)/high-risk (5: 27.9%) class according to EU-TIRADS and 30.9% according to Kwak-TIRADS (4B: 22.1%, 4C and 5: 8.8%). This proportion further increased to 63.3% for EU-TIRADS (4: 22.1%, 5: 41.2%) or 51.5% for Kwak-TIRADS (4B: 33.8%, 4C and 5: 17.7%) at FU1 and 75% (4: 29.4%%, 5: 45.6%%) or 58.8% (4B: 36.8%, 4C and 5: 22.1%) at FU 2, respectively (Figure 1). The proportion of AFTNs that were sonographically low-risk TNs before RIT (69.1% for Kwak-TIRADS and 52.9% for EU-TIRADS) decreased to 41.2% for Kwak-TIRADS (EU-TIRADS 25%) during the FU. Figure 2 and Figure 3 show examples of two AFTNs before and after RIT.

## 4. Discussion

We described the morphological changes of solitary AFTNs after RIT using standardized US. As a consequence of the RIT, the nodules’ characteristics changed towards a more hypoechogenic appearance compared to the surrounding thyroid tissue. The solid proportion of the nodules increased, and they appeared less cystic. Calcifications were observed more frequently after RIT. Interestingly, as a consequence of the morphological changes caused by RIT, the AFTNs more often developed blurred rather than irregular margins, of which the former is a typical finding for malignant TNs [15,16,17]. It is also noteworthy that only a few AFTNs showed a taller-than-wide shape, which is also a highly specific US feature for malignancy [20]. This feature was apparently not affected by RIT. Nevertheless, the findings of our study indicate that AFTNs treated by RIT can mimic suspicious lesions in US imaging. However, in a previous study by our group, we showed that AFTNs can also present with suspicious features on US before RIT [3]. These results were confirmed by our present study. Almost 50% of the AFTNs were classified as intermediate- or high-risk TNs before RIT when using EU-TIRADS. This fact underlines the importance of thyroid scintigraphy to exclude malignancy.

There is a very limited data on the systematic examination of morphological changes in AFTNs after RIT. Only one single study in patients with Graves’ disease described a relationship between changes of echogenicity and RIT [13]. In detail, a RIT causes an increase of inhomogeneity and a decrease of echogenicity of thyroid tissue—similar to our results for treated nodules. The resulting hypoechoic appearance may be due to radiation-induced conversion to scar tissue.

Marković and Eterović described in their analysis of patients with Graves’ disease that the response rate after RIT is higher for pretherapeutically described hypoechoic thyroid glands than normoechoic thyroid glands [12]. To our knowledge, there are no studies linking the treatment response rate to the morphological features and their changes in AFTNs before and after RIT. This aspect might be interesting for future prospective studies. Moreover, there is also a lack of knowledge regarding whether there is a connection between the achieved radiation dose and specific morphological changes of AFTNs. On the other hand, it can be hypothesized that certain morphological features may trigger a different treatment approach or target dose. For local ablative procedures (for example, radio frequency ablation (RFA)), such connections between response and morphological changes have already been investigated. Solid nodules showed a lower reduction of volume compared to mixed cystic/solid or predominantly cystic nodules [21,22,23].

Our study shows some limitations. All the AFTNs were retrospectively stratified by one experienced nuclear medicine physician (S.S.), while different physicians from two institutions performed the US examinations at their own site. A pertinent investigation of the interobserver variability at the two sites was not conducted. However, despite increasing experience and regular training on US reporting systems, it cannot be ruled out that relevant US criteria were not documented and led to an incorrect classification. Moreover, the number of patients in our study was not very high. Therefore, prospective studies with a higher number of patients and prospective TIRADS classifications are needed and already planned. At both study sites, the achieved dose of the RIT differed significantly, which can be explained by institution-specific treatment protocols. Additionally, follow-up data were obtained from clinical routines. Therefore, the time of follow-up consultation varied within a few months. While the first follow-up was performed in a relatively narrow time period after RIT (3–7 months), the FU2 examination had a larger time range (11–31 months).

## 5. Conclusions

A significant percentage of AFTNs presented as suspicious for malignancy according to TIRADS before RIT, and this number increased significantly after therapy on follow-up. Physicians should be aware of this result when performing ultrasound in this group of patients to avoid unnecessary further interventions.

## Figures and Tables

**Table 1:** Clinical characteristics of the patient collectives.

	All	Study Site 1 Hanau	Study Site 2 Magdeburg	*p*
Patients	68	34	34	
Age (years) mean ± SD	60.6 ± 13.0	58.8 ± 14.3	62.5 ± 11.4	0.228 *
Sex Female Male	39 29	21 13	18 16	0.624 ^†^
Location AFTN Right lobe Left lobe Isthmus	35 28 5	14 16 4	21 12 1	0.167 ^†^
Max. size AFTN (mm) median (25/75th-P)	33 (27/36)	32 (24.8/35)	33 (27/38.3)	0.144 ^‡^
Activity (MBq) median (25/75th-P)	539 (347/787.5)	534.5 (403.5/743.8)	539 (320.8/807.5)	0.806 ^‡^
Dose AFTN (Gy) median (25/75th-P)	361.5 (202.3/422)	398.5 (277.8/521.8)	241.5 (151.3/392)	0.0004 ^‡^

* Student’s *t*-test; ^†^ Fisher’s exact test; ^‡^ Wilcoxon’s rank sum test; SD standard deviation; AFTN autonomously functioning thyroid nodule.

**Table 2:** US imaging features before RIT and at the FU for all AFTNs.

Ultrasound Features	Before RIT	FU1 5 months	FU2 16 months	*p*-Value * (pre-RIT to FU1)	*p*-Value * (pre-RIT to FU2)
Composition, *n* (%)				0.09	0.28
solid/<10% cystic	32 (47.06)	38 (55.88)	41 (60.29)		
10–50% cystic	32 (47.06)	21 (30.88)	23 (33.82)		
>50% cystic	4 (5.88)	9 (13.24)	4 (5.88)		
Calcifications, *n* (%)				0.23	0.37
none	53 (77.94)	49 (72.06)	44 (64.71)		
macrocalcifications	7 (10.29)	3 (4.41)	9 (13.24)		
microcalcifications	5 (7.35)	11 (16.18)	9 (13.24)		
both	3 (4.41)	5 (7.35)	6 (8.82)		
Margin, *n* (%)				0.08	0.002
well-circumscribed	61 (89.71)	54 (79.41)	45 (66.18)		
irregular	1 (1.47)	7 (10.29)	5 (7.35)		
blurred	6 (8.82)	7 (10.29)	18 (26.47)		
Shape, *n* (%)				0.45	0.45
wider-than-tall	57 (83.82)	61 (89.71)	61 (89.71)		
taller-than-wide	11 (16.18)	7 (10.29)	7 (10.29)		
Echogenicity, *n* (%)				0.05	0.003
hypoechoic	21 (30.88)	30 (44.12)	34 (50)		
isoechoic	47 (69.12)	35 (51.47)	30 (44.12)		
hyperechoic	0 (0)	2 (2.94)	0 (0)		
marked-hypoechoic	0 (0)	1 (1.47)	4 (5.88)		

* Fisher’s exact test; US ultrasound; RIT radioiodine therapy; FU follow-up; AFTN autonomously functioning thyroid nodule.

## References

[1] Giovanella L., Avram A.M., Iakovou I., Kwak J., Lawson S.A., Lulaj E., Luster M., Piccardo A., Schmidt M., Tulchinsky M. (2019). EANM practice guideline/SNMMI procedure standard for RAIU and thyroid scintigraphy. Eur. J. Nucl. Med. Mol. Imaging.

[2] Reschini E., Ferrari C., Castellani M., Matheoud R., Paracchi A., Marotta G., Gerundini P. (2006). The trapping-only nodules of the thyroid gland: Prevalence study. Thyroid.

[3] Schenke S., Seifert P., Zimny M., Winkens T., Binse I., Goerges R. (2018). Risk stratification of thyroid nodules using Thyroid Imaging Reporting And Data System (TIRADS): The omission of thyroid scintigraphy increases the rate of falsely suspected lesions. J. Nucl. Med..

[4] Ruhlmann M., Stebner V., Görges R., Farahati J., Simon D., Bockisch A., Rosenbaum-Krumme S., Nagarajah J. (2014). Diagnosis of hyperfunctional thyroid nodules: Impact of US-elastography. Nuklearmedizin.

[5] Giovanella L., Piccardo A., Pezzoli C., Bini F., Ricci R., Ruberto T., Trimboli P. (2018). Comparison of high intensity focused ultrasound and radioiodine for treating toxic thyroid nodules. Clin. Endocrinol..

[6] Ronga G., Filesi M., D’Apollo R., Toteda M., Di Nicola A.D., Colandrea M., Travascio L., Vestri A.R., Montesano T. (2013). Autonomous functioning thyroid nodules and 131I in diagnosis and therapy after 50 years of experience: What is still open to debate?. Clin. Nucl. Med..

[7] Dietlein M., Grünwald F., Schmidt M., Schneider P., Verburg F.A., Luster M. (2016). Radioiodine therapy for benign thyroid diseases (version 5). German Guideline. Nuklearmedizin.

[8] Stokkel M., Handkiewicz Junak D., Lassmann M., Dietlein M., Luster M. (2010). EANM procedure guidelines for therapy of benign thyroid disease. Eur. J. Nucl. Med. Mol. Imaging.

[9] Cervelli R., Mazzeo S., Boni G., Boccuzzi A., Bianchi F., Brozzi F., Santini P., Vitti P., Cioni R., Caramella D. (2019). Comparison between radioiodine therapy and single-session radiofrequency ablation of autonomously functioning thyroid nodules: A retrospective study. Clin. Endocrinol..

[10] Taratini B., Ciuoli C., Di Cairano D., Guarino E., Mazzucato P., Montanaro A., Burroni L., Vattimo A., Pacini F. (2006). Effectiveness of radioiodine (131-I) as definitive therapy in patients with autoimmune and non-autoimmune hyperthyroidism. J. Endocrinol. Investig..

[11] Erdoğan M.F., Küçük N.O., Anil C., Aras S., Ozer D., Aras G., Kamel N. (2004). Effect of radioiodine therapy on thyroid nodule size and function in patients with toxic adenomas. Nucl. Med. Commun..

[12] Markovic V., Eterovic D. (2007). Thyroid echogenicity predicts outcome of radioiodine therapy in patients with Graves’ disease. J. Clin. Endocrinol. Metab..

[13] English C., Casey R., Bell M., Bergin D., Murphy J. (2016). The Sonographic Features of the Thyroid Gland After Treatment with Radioiodine Therapy in Patients with Graves’ Disease. Ultrasound Med. Biol..

[14] Horvath E., Majlis S., Rossi R., Franco C., Niedmann J.P., Castro A., Dominguez M. (2009). An ultrasonogram reporting system for thyroid nodules stratifying cancer risk for clinical management. J. Clin. Endocrinol. Metab..

[15] Kwak J.Y., Han K.H., Yoon J.H., Moon H.J., Son E.J., Park S.H., Jung H.K., Choi J.S., Kim B.M., Kim E.-K. (2011). Thyroid imaging reporting and data system for US features of nodules: A step in establishing better stratification of cancer risk. Radiology.

[16] Russ G., Bonnema S.J., Erdogan M.F., Durante C., Ngu R., Leenhardt L. (2017). European Thyroid Association Guidelines for Ultrasound Malignancy Risk Stratification of Thyroid Nodules in Adults: The EU-TIRADS. Eur. Thyroid J..

[17] Tessler F.N., Middleton W.D., Grant E.G., Hoang J.K., Berland L.L., Teefey S.A., Cronan J.J., Beland M.D., Desser T.S., Frates M.C. (2017). ACR Thyroid Imaging, Reporting and Data System (TI-RADS): White Paper of the ACR TI-RADS Committee. J. Am. Coll. Radiol..

[18] Schenke S., Rink T., Zimny M. (2015). TIRADS for sonographic assessment of hypofunctioning and indifferent thyroid nodules. Nuklearmedizin.

[19] Schenke S., Zimny M. (2018). Combination of Sonoelastography and TIRADS for the Diagnostic Assessment of Thyroid Nodules. Ultrasound Med. Biol..

[20] Remonti L.R., Kramer C.K., Leitão C.B., Pinto L.C.F., Gross J.L. (2015). Thyroid ultrasound features and risk of carcinoma: A systematic review and meta-analysis of observational studies. Thyroid.

[21] Kim Y.-S., Rhim H., Tae K., Park D.W., Kim S.T. (2006). Radiofrequency ablation of benign cold thyroid nodules: Initial clinical experience. Thyroid.

[22] Lim H.K., Lee J.H., Ha E.J., Sung J.Y., Kim J.K., Baek J.H. (2013). Radiofrequency ablation of benign non-functioning thyroid nodules: 4-year follow-up results for 111 patients. Eur. Radiol..

[23] Aysan E., Idiz U.O., Akbulut H., Elmas L. (2016). Single-session radiofrequency ablation on benign thyroid nodules: A prospective single center study: Radiofrequency ablation on thyroid. Langenbecks Arch. Surg..

